# Supplementary material for: DRB1, DRB2 and DRB4 Are Required for Appropriate Regulation of the microRNA399/PHOSPHATE2 Expression Module in Arabidopsis thaliana
Source: Plants (Basel). 2019 May 13;8(5):124. doi: 10.3390/plants8050124 (PMC6571617; doi:10.3390/plants8050124)
Supplement: Supplementary file 1 [file plants-08-00124-s001.pdf]

DRB1, DRB2 and DRB4 are required for appropriate regulation of the microRNA399/ *PHOSPHATE2* expression module in *Arabidopsis thaliana*

Joseph L Pegler[1], Jackson MJ Oultram[1], Christopher PL Grof[1] and Andrew L Eamens[1,*]

[1] Centre for Plant Science, School of Environmental and Life Sciences, Faculty of Science, University of Newcastle, Callaghan 2308, New South Wales, Australia

* Correspondence: E-mail: andy.eamens@newcastle.edu.au; Tel.: +61-249-217-784

Supplemental Table S1: Sequences of the DNA oligonucleotides used in this study.

The DNA oligonucleotide sequence of the stem-loop primer used to prime the reverse transcription of a miR399-specific cDNA is provided in the below Table (denoted by RTSL). Post synthesis of a miR399-specific cDNA, the miR399-specific forward primer (denoted by RTF in the below Table) and a generic reverse primer (denoted by SLR in the below table (underlined sequence of the RTSL primer identifies the binding site for the generic reverse primer)) were used to quantify miR399 abundance. Also provided in the below Table is the sequence of the DNA oligonucleotide used as either the forward (denoted by RTF in the below Table) or reverse (denoted by RTR in the below Table) primer to quantify the expression of a cohort of PO_4-related high molecular weight RNA transcripts. The snoRNA, *snoR101*, was used to normalize the abundance of the miR399 across the different plant lines, tissues and growth regimes assessed in this study. The reference gene, *UBI10* (*AT4G05320*), was used to normalise the expression of each transcript belonging to the cohort of PO4-related high molecular weight RNA transcripts assessed in this study.

Targeted Sequence	Primer Name	Oligonucleotide sequence (5' to 3')
**DNA oligonucleotides used for miR399-specific cDNA synthesis and RT-qPCR abundance quantification**		
**miR399**	**p399-RTSL**	GTCGTATCCAGTGCAGGGTCCGAGGTATTCGCACTGGATACGACCAGGGC
	**p399-RTF**	GCATGCCAAAGGAGATTTGCCCTG
**miRNA stem-loop oligo**	**pSLR-Generic**	CCAGTGCAGGGTCCGAGGTA
**snoR101**	**pSnoR-RTF**	CTTCACAGGTAAGTTCGCTTG
	**pSnoR-RTR**	AGCATCAGCAGACCAGTAGTT
**DNA oligonucleotides used for the analysis of expression of a cohort of high molecular weight transcripts**		
***UBI10* (*AT4G05320*)**	**pUBI10-RTF**	GGCCTTGTATAATCCCTGATGAATAAG
	**pUBI10-RTR**	AAAGAGATAACAGGAACGGAAACATA
***PHO2* (*AT2G33770*)**	**pPHO2-RTF**	ACCGTTTCTCATCAAGGCGT
	**pPHO2-RTR**	GTGCCCGTCCACCATAAGAA
***IPS1* (*AT3G09922*)**	**pIPS1-RTF**	AAATGGCCATCCCCTAGC
	**pIPS1-RTR**	GCCATCCCCTAAAAGATGAA
***MIR399A* (*AT1G29265*)**	**p399A-RTF**	AGGGTAAGATCTCTATTGGCAGGAAAC
	**p399A-RTR**	GCAGAAGAATTACAGGGCAAATCTCC

MIR399B	**p399B-RTF**	TCTCCATTGGCAGGTCCTTTACTTCC
	**p399B-RTR**	TCAGGGCAACTCTCCTTTGGCAG
MIR399C	**p399C-RTF**	CATCTTTCTATTGGCAGGCGACTTGG
	**p399C-RTR**	AAGCAGTGACAGGGCAACTCTCC
MIR399D	**p399D-RTF**	AATACTCCTATGGCAGATCGCATTGG
	**p399D-RTR**	TCCTTTGGCAGAGAAGCATTTTACTTG
MIR399E	**p399E-RTF**	AAGCATTACAGGGCGAATCCTC
	**p399E-RTR**	CGAAGCATTGCGAGGCAAATCTCCA
MIR399F	**p399F-RTF**	GCATTACAGGGCAAGATCACCATTGG
	**p399F-RTR**	GCGCAAGAGAATTACCGGGCAAATC
PHR1	**pPHR1-RTF**	AAACCAACCCGGCGATTCA
	**pPHR1-RTF**	CAGCCCATTCATGCCAATCACTT
PHT1;4	**pPHT14-RTF**	TGTGCCGGCCGAAATCT
	**pPHT14-RTR**	TTGCTCCTAATTTTCCTGATGCT
PHT1;8	**pPHT18-RTF**	CCCGAAGTAAACCGTATGAGAA
	**pPHT18-RTR**	AATACGTCACCAAGATTCCAGCAA
PHT1;9	**pPHT19-RTF**	TGGAGCTGCAGGGAAGTTTG
	**pPHT19-RTR**	ATCTGGAAAACCGTCCTCTTCAT
